# Relationship between cognitive functions and empathy in patients with neurocognitive deficit

**DOI:** 10.1192/j.eurpsy.2021.1111

**Published:** 2021-08-13

**Authors:** D. Galletta, I. Lauria, A.M. Mastrola, M. Confuorto

**Affiliations:** Department Of Head-neck Care Unit Of Psychiatry And Psychology “federico Ii” University Hospital Naples, “Federico II” University Hospital Naples, Italy, Naples, Italy

**Keywords:** cognitive functions, empathy, perspective taking, neurocognitive deficit

## Abstract

**Introduction:**

Empathy is a social emotive skill that let to experience the same feelings of another person without being in the same situation. It changes during the growth becoming more over sophisticated with the involving of cognitive functions such as perspective taking (Hoffmann, 2000). Several researches observed a correlation between empathy and psychopathologies that involve cognitive functions such as attention and executive functions (Abdel-Hamid et al., 2019; Blair, 2018; Pijper et al., 2018) or decision-making (Francis et al., 2019).

**Objectives:**

To investigate the impact of cognitive impairment on different empathy dimensions.

**Methods:**

80 subjects with severe neurocognitive deficit were examined. WAIS-R, neuropsychological battery and IRI test were performed.

**Results:**

The impairment of perspective-taking dimension was significantly noticeable (=or<17/30). In addition, impairments of self-regulation process and inner-state monitoring mechanisms were also observed (=or<18/40).

**Conclusions:**

According to previous researches, this study confirms that empathy can be reduced when cognitive functions are compromised by psychopathologies or other medical conditions. Personal distress and perspective taking are empathy dimensions more affected in these cases.

